# The Evolving Therapeutic Landscape of Gallic Acid: A Review of Mechanistic Insights and Clinical Potential

**DOI:** 10.1002/mnfr.70491

**Published:** 2026-05-08

**Authors:** Woo Hyun Park

**Affiliations:** ^1^ Department of Physiology, Medical School Jeonbuk National University Jeonju Republic of Korea

**Keywords:** bioactive materials, gallic acid, metabolic disease, NLRP3 inflammasome, oncology, redox duality

## Abstract

Gallic acid (GA), a plant‐derived phenolic compound, is evolving from a general antioxidant into a specific molecular modulator and a functional building block for advanced biomedical materials. This review synthesizes recent insights underpinned by GA's “redox duality”—its capacity to act as either a radical scavenger or a pro‐oxidant. In metabolic disorders, GA reprograms cellular machinery by modulating the miR‐709/Nrf2 axis, enhancing LDL clearance via LDLR/PCSK9, and targeting the incretin system. In oncology, GA exhibits context‐dependent toxicity, acting as a selective pro‐oxidant to induce apoptosis and synergizing with tyrosine kinase inhibitors (TKIs), though safety interactions require careful assessment. GA's expanded neuropharmacological profile includes mitigating ferroptosis via GPX4 rescue and acting as a positive allosteric modulator of Kv1.1 channels. It also potently inhibits the NLRP3 inflammasome in gouty arthritis. Beyond traditional pharmacology, recent studies highlight GA's emergence in material science, including the development of GA‐based Metal‐Organic Frameworks (MOFs) and functional hydrogels for osteoarthritis, kidney stone prevention, and wound healing. Despite broad bioactivity, poor bioavailability remains a significant challenge, necessitating innovative bioactive material design and rigorous clinical validation.

## Graphical abstract

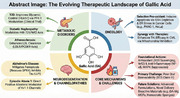

**Abbreviations**
AD: Alzheimer's Disease
Akt: protein kinase B
AMPK: AMP‐activated protein kinase
ARE: antioxidant Response Element
Aβ: amyloid‐beta
BDE: bond Dissociation Enthalpy
BCS: biopharmaceutics Classification System
CAT: catalase
CML: chronic myeloid leukemia
COX‐2: cyclooxygenase‐2
DFT: density functional theory
DM: diabetes mellitus
DN: diabetic nephropathy
DPP‐IV: dipeptidyl peptidase‐IV
EA1: episodic ataxia 1
EGFR: epidermal growth factor receptor
ER: endoplasmic reticulum
ERK1/2: extracellular signal‐regulated kinase
FOXO3: forkhead box O3
GA: gallic acid
GA‐Mg: gallic acid‐magnesium
GLP‐1: glucagon‐like peptide‐1
GLUT4: glucose transporter type 4
GPX: glutathione peroxidase
GPX4: glutathione peroxidase 4
GSH: glutathione
HAT: hydrogen atom transfer
HMGB1: high‐mobility group box 1
HNF1α: hepatocyte nuclear factor‐1α
IL‐6: interleukin‐6
iNOS: inducible nitric oxide synthase
Kv1.1: voltage‐gated K+ channel subtype 1.1
LDL: low‐density lipoprotein
LDLR: low‐density lipoprotein receptor
LLPS: liquid‐liquid phase separation
MAPK: mitogen‐activated protein kinase
MMPs: matrix metalloproteinases
MOF: metal‐organic framework
MPNs: metal‐phenolic networks
NDDS: novel drug delivery systems
NFTs: neurofibrillary tangles
NF‐κB: nuclear factor‐kappa B
NLRP3: NLR family pyrin domain containing 3
Nrf2: nuclear factor erythroid 2‐related factor 2
OA: osteoarthritis
PCSK9: proprotein convertase subtilisin/kexin type 9
PD: Parkinson's Disease
PFK‐1: phosphofructokinase‐1
RNS: reactive nitrogen species
ROS: reactive oxygen species
SOD: superoxide dismutase
T2D: type 2 diabetes
TKI: tyrosine kinase inhibitor
TNF‐α: tumor necrosis factor‐alpha
VSD: voltage‐sensing domain

## 1 Introduction: Gallic Acid as a Pleiotropic Phytochemical

### 1.1 Defining Gallic Acid in the Pharmacological Context

Nature provides a vast repository of bioactive compounds, many of which have served as the foundation for modern medicine. Among these, gallic acid (GA), or 3,4,5‐trihydroxybenzoic acid, has emerged as a phytochemical of exceptional interest [1]. It is a naturally occurring phenolic acid widely distributed throughout the plant kingdom, found in both its free form and as a fundamental structural unit of more complex polyphenols, particularly hydrolyzable tannins known as gallotannins [1, 2]. For centuries, plants rich in GA have been used in traditional medicine [3], a practice now being validated by rigorous scientific investigation, which has confirmed a wide range of medicinal properties, including anti‐inflammatory and antineoplastic effects [4]. Among these, its hepatoprotective activity is particularly well‐documented; for example, a recent review by Chen et al. [5] synthesized data from numerous animal models (e.g., CCl_4_‐ or alcohol‐induced liver injury in rodents) showing GA's ability to protect hepatocytes by mitigating oxidative stress, inhibiting inflammatory cascades, and preventing apoptosis.

Contemporary research has established GA as a molecule with extensive health benefits, underpinned by a remarkable range of biological activities [6]. Its potent antioxidant, anti‐inflammatory, antimicrobial, antiviral, and antineoplastic properties are well‐documented [1, 4, 7]. Oxidative stress, an imbalance resulting from the overproduction of damaging free radicals, is a key driver of numerous human diseases, including atherosclerosis, cancer, neurodegeneration, and cardiovascular disorders. This link is well‐established in cardiovascular disease [8] and in the context of diabetes and its complications, particularly in an obese state where inflammation and oxidative stress are pathologically linked [9, 10]. In this context, naturally occurring antioxidants like GA are considered highly promising for new drug development due to their capacity to prevent or mitigate this oxidative damage [4, 10]. This preventive role has positioned the GA nucleus as an “indispensable anchor for designing” novel pharmacological agents, making it a critical scaffold in modern medicinal chemistry [4].

### 1.2 Chemical Architecture and Biosynthesis

**FIGURE 1:**
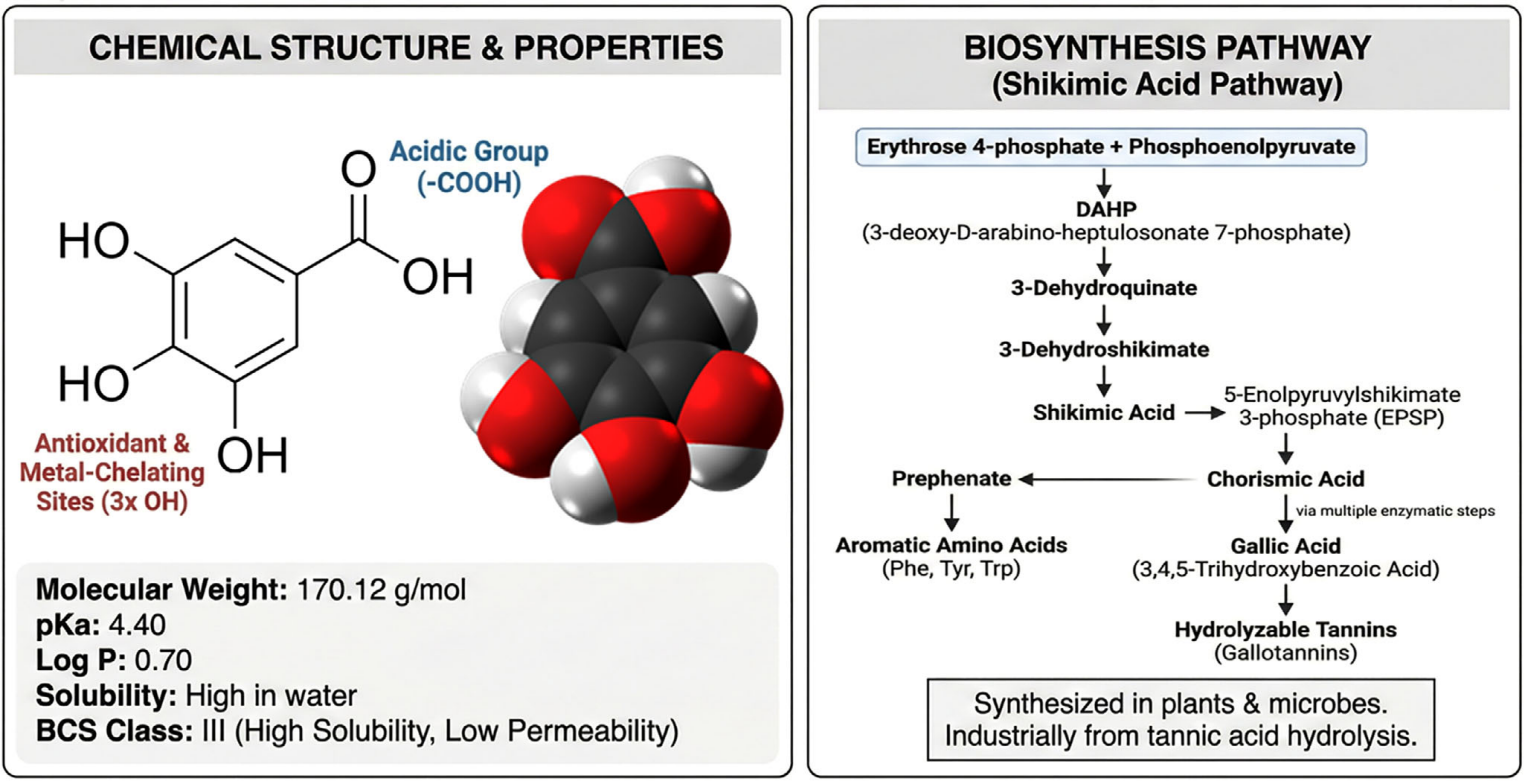
Profile of gallic acid: chemical structure, physicochemical properties, and biosynthesis pathway. The left panel presents the 2D chemical structure and a space‐filling model of gallic acid (C_7_H_6_O_5_). Key functional groups are indicated: the three hydroxyl groups (3x OH) responsible for its potent antioxidant and metal‐chelating activities, and the carboxyl group (─COOH) conferring acidity. Below the structure, essential physicochemical parameters are summarized, including molecular weight (170.12 g/mol), pKa (4.40), Log P (0.70), high water solubility, and its classification as a BCS Class III compound. The right panel illustrates the Shikimic Acid biosynthesis pathway, tracing the enzymatic conversion of the precursors Erythrose 4‐phosphate and Phosphoenolpyruvate. The diagram details the pathway through Shikimic Acid to Chorismic Acid, depicting the bifurcation towards aromatic amino acids or gallic acid (via multiple enzymatic steps). Further, the diagram depicts the production of gallic acid via the enzymatic hydrolysis of hydrolyzable tannins (gallotannins), indicating its natural synthesis in plants and microbes as well as its industrial production from tannic acid hydrolysis. Notably, recent advancements have also enabled efficient biosynthesis using syntrophic *E. coli* co‐culture systems.

The profound biological activity of GA (molecular formula C_7_H_6_O_5_) is a direct consequence of its unique chemical architecture. Its structure consists of a benzene ring with a carboxyl group (─COOH) and, critically, three adjacent hydroxyl (─OH) groups at the 3, 4, and 5 positions (Figure 1). This specific arrangement is the primary determinant of its potent antioxidant and metal‐chelating capabilities [1, 11]. As recently elucidated by Park [12], this structure underpins a fundamental ‘redox duality,’ allowing GA to function either as a radical scavenger or, under specific conditions, as a pro‐oxidant via Fenton‐type reactions. This dual nature is the primary conceptual framework for its functional versatility, ranging from antioxidant protection to selective cytotoxicity [12].

In nature, GA is synthesized in plants and microorganisms through the shikimic acid pathway (Figure 1), while industrially, it is most commonly produced through the enzymatic hydrolysis of tannic acid [1]. Recently, metabolic engineering has expanded production possibilities; Wang et al. [13] achieved efficient biosynthesis of GA using a syntrophic *Escherichia coli* co‐culture system, offering a sustainable alternative to traditional extraction methods. Its physicochemical properties (pKa 4.40, Log P 0.70) and high water solubility classify it as a Biopharmaceutics Classification System (BCS) Class III compound, a key factor that, as will be discussed, critically impacts its pharmacokinetic profile [1].

GA is ubiquitous in the human diet, a topic of increasing interest in nutritional science [14]. It is abundant in beverages like tea and red wine, and in foods such as grapes, walnuts, pomegranates, and strawberries [1, 2, 15]. As highlighted in a 2024 review by Hadidi et al., this widespread dietary availability positions GA as a significant contributor to the health benefits associated with plant‐based diets. Ultimately, GA's structure—combining a planar aromatic ring, an acidic carboxyl group, and three vicinal hydroxyls—is perfectly suited for donating electrons to neutralize free radicals and forming stable complexes with metal ions, providing the fundamental basis for its biological effects.

### 1.3 Literature Search Methodology

To provide a focused synthesis of recent mechanistic advancements, this review was prepared based on a targeted literature search. Key databases, including PubMed, Scopus, and Google Scholar, were queried for articles published primarily between 2015 and 2025. Search terms included “gallic acid” in combination with “mechanism”, “oncology”, “diabetes”, “neurodegeneration”, “pharmacokinetics”, “channelopathy”, “ferroptosis”, and “drug delivery”. Inclusion criteria prioritized recent (post‐2020) original research articles and comprehensive reviews that elucidated specific molecular pathways, novel therapeutic targets, or advanced formulation strategies. Foundational papers and highly cited earlier reviews were included for essential context.

### 1.4 Distinguishing Scope of this Review

The pharmacological properties of GA have been the subject of several excellent reviews. For instance, Badhani et al. [6] provided a comprehensive overview of its dual antioxidant/pro‐oxidant potential, while Kahkeshani et al. [7] systematically reviewed its effects in various diseases, correctly noting at the time that evidence was limited to cellular and animal studies. More recent reviews have focused on specific pathological processes, such as its role in obesity‐related inflammation [9], its general anti‐inflammatory mechanisms [16], or its broad health benefits and food applications [14].

This review seeks to build upon that foundational work by providing a distinct contribution. Its primary focus is to synthesize the most recent and emergent mechanistic discoveries that are repositioning GA from a general “nutraceutical” to a highly specific “molecular modulator”. The unique scope of this article is its emphasis on novel, protein‐specific interactions and pathway modulations that have been elucidated primarily in the last 3–5 years. Key areas of focus that distinguish this review include as follows:

1. Advanced neuropharmacology: A detailed exploration of GA's role in inhibiting ferroptosis (via GPX4) and pathological liquid‐liquid phase separation (LLPS), and its paradigm‐shifting discovery as a direct allosteric modulator of Kv1.1 ion channels.
2. Specific metabolic reprogramming: A mechanistic dive into how GA modulates complex pathways beyond general antioxidant effects, such as the miR‐709/Nrf2 axis in diabetic nephropathy and the LDLR/PCSK9 axis in cholesterol regulation.
3. Modern therapeutic strategies: A forward‐looking perspective on how advanced formulations, novel drug delivery systems (NDDS), and GA‐based biomaterials are being designed to overcome their well‐known pharmacokinetic limitations.
4. By concentrating on these recent, specific, and advanced molecular insights, this review aims to provide an updated perspective on GA's evolving therapeutic landscape and future clinical potential.

## 2 The Mechanistic Cornerstone: Antioxidant and Anti‐Inflammatory Actions

GA's therapeutic effects are largely rooted in its ability to modulate two fundamental, interconnected pathological processes: oxidative stress and inflammation [16]. GA acts as an upstream regulator, intervening at the source of cellular damage.

### 2.1 Molecular Basis of Antioxidant Activity

GA combats oxidative stress through a multi‐pronged strategy. First, it is a potent free radical scavenger. The three phenolic hydroxyl groups are excellent hydrogen/electron donors, allowing GA to directly neutralize superoxide radicals (O_2_•^−^), hydroxyl radicals (•OH), and peroxynitrite (ONOO^−^) [10]. Recent quantum chemical studies utilizing density functional theory (DFT) have provided a 21st‐century molecular explanation for this efficiency. Spiegel et al. [17] calculated the Bond Dissociation Enthalpy (BDE) of phenolic compounds, confirming that GA's hydroxyl groups possess exceptionally low BDE values. This thermodynamic property facilitates rapid hydrogen atom transfer (HAT) to free radicals, making GA kinetically superior to many other polyphenols. A comprehensive study by Kim and Lee [11] using DPPH and other scavenging assays definitively correlated this high activity with the 3,4,5‐trihydroxy structure, establishing it as more potent than other phenolics like quercetin or catechin on a molar basis.

Second, GA functions as a powerful metal chelator. The adjacent hydroxyl groups form stable coordination complexes with transition metals like iron (Fe^2^^+^/Fe^3^^+^) and copper (Cu^+^/Cu^2^^+^), sequestering them and preventing their participation in radical‐generating Fenton reactions [1]. This mechanism was recently exploited by Santoso et al. [18] in the design of GA‐based metal‐phenolic networks, which rely on this specific, high‐affinity chelation.

Third, GA reinforces the body's endogenous antioxidant defense systems. It boosts the cell's innate capacity to handle oxidative stress by activating the Nuclear factor erythroid 2‐related factor 2 (Nrf2) pathway. As demonstrated by Lee et al. [19] in a mouse model of diabetic nephropathy, GA treatment (50 mg/kg) restored the activity of key antioxidant enzymes, including superoxide dismutase (SOD), catalase (CAT), and glutathione peroxidase (GPX). This effect was directly linked to GA's ability to promote Nrf2 nuclear translocation, which in turn upregulates the genes for these protective enzymes. Concurrently, GA helps replenish the intracellular pool of glutathione (GSH), the cell's most abundant non‐enzymatic antioxidant [10].

### 2.2 Attenuation of Inflammatory Pathways

Oxidative stress and inflammation are deeply intertwined in a vicious cycle. GA effectively disrupts this cycle by targeting key inflammatory pathways, a mechanism thoroughly reviewed by Bai et al. [16].

GA suppresses the production of critical pro‐inflammatory mediators. In various cellular models (e.g., LPS‐stimulated macrophages), GA (at concentrations typically ranging from 10–100 µM) has been shown to inhibit the expression and activity of inducible nitric oxide synthase (iNOS) and cyclooxygenase‐2 (COX‐2), thereby reducing the production of nitric oxide (NO) and pro‐inflammatory prostaglandins [10].

More profoundly, GA modulates the intracellular signaling cascades that orchestrate the inflammatory response. A primary target is the nuclear factor‐kappa B (NF‐κB) pathway, a master regulator of inflammatory gene expression. As summarized by Seo et al. [20], GA inhibits the activation of NF‐κB, often by preventing the phosphorylation and degradation of its inhibitor, IκBα. This blocks the nuclear translocation of the p65 subunit, thereby preventing the transcription of cytokines like tumor necrosis factor‐alpha (TNF‐α) and interleukin‐6 (IL‐6). Additionally, GA modulates other critical routes, including the mitogen‐activated protein kinase (MAPK) pathways (specifically p38, ERK1/2) and the pro‐survival protein kinase B (Akt) pathway, which are often dysregulated in inflammatory conditions [10, 16].

**FIGURE 2:**
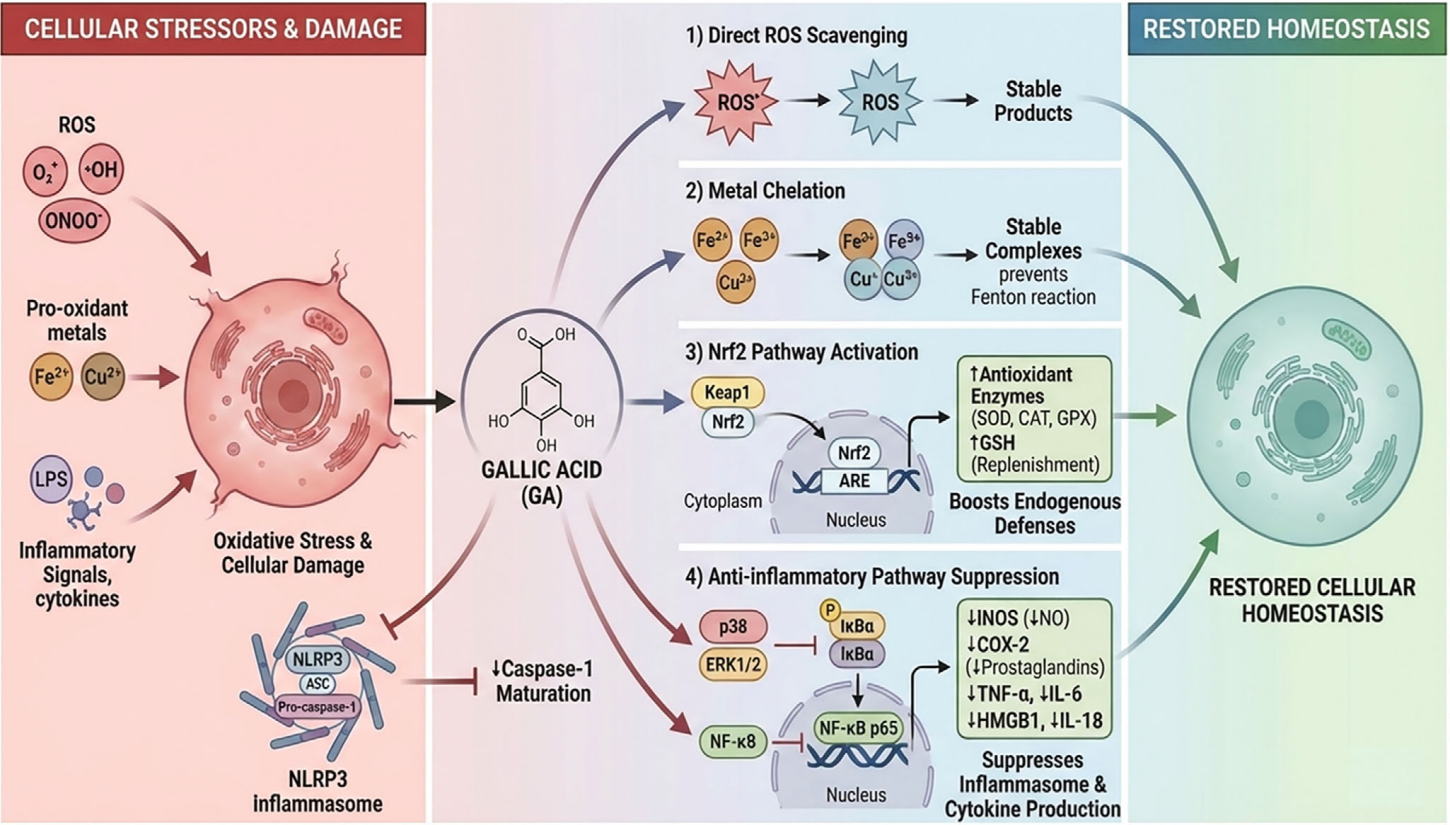
Core antioxidant and anti‐inflammatory pathways modulated by gallic acid (preclinical models). This schematic illustrates the multifaceted mechanisms by which gallic acid (GA) mitigates cellular stress to restore homeostasis. The left panel (“cellular stressors and damage”) depicts various insults, including reactive oxygen species (ROS), pro‐oxidant metals, and inflammatory signals (e.g., LPS), leading to oxidative stress. The central panels detail four key therapeutic interventions driven by GA: (1) Direct ROS Scavenging: GA directly neutralizes harmful ROS into stable products. (2) Metal chelation: GA sequesters transition metals (Fe^2^^+^, Cu^2^^+^), forming stable complexes and preventing Fenton reaction‐mediated radical generation. (3) Nrf2 pathway activation: GA promotes the nuclear translocation of Nrf2 by dissociating it from Keap1. Nrf2 binds to the antioxidant response element (ARE) to boost endogenous defenses through the upregulation of antioxidant enzymes (SOD, CAT, GPX) and glutathione (GSH) replenishment. (4) Anti‐inflammatory pathway suppression: GA exerts broad anti‐inflammatory effects by inhibiting MAPK (p38, ERK1/2) and NF‐κB signaling. Furthermore, GA suppresses the NLRP3 inflammasome and caspase‐1 maturation. These actions collectively reduce the production of inflammatory mediators (iNOS, COX‐2, TNF‐α, IL‐6, HMGB1, IL‐18). The convergence of these protective mechanisms leads to “restored cellular homeostasis,” as shown in the right panel.

Beyond the canonical NF‐κB pathway, recent investigations have highlighted GA's specific modulation of the NLRP3 inflammasome, a multiprotein oligomer critical for innate immune activation. Lin et al. [21] demonstrated that GA effectively inhibits the assembly and activation of the NLRP3 inflammasome in a mouse model of gouty arthritis. Mechanistically, this suppression reduces the maturation of caspase‐1 and the subsequent release of high‐mobility group box 1 (HMGB1) and pro‐inflammatory cytokines such as IL‐1β and IL‐18. By targeting this upstream cytosolic sensor, GA offers a more comprehensive anti‐inflammatory blockade than previously understood. These core mechanisms are visually summarized in Figure 2.

## 3 Gallic Acid in the Management of Metabolic Disorders

GA demonstrates a remarkable capacity to intervene in specific metabolic pathways central to diseases like diabetes and atherosclerosis, acting as an active molecular agent that can reprogram pathological metabolic circuits.

### 3.1 Combating Diabetes Mellitus and Its Complications

In diabetes (DM), oxidative stress and inflammation are key drivers of hyperglycemia and insulin resistance [9, 10]. GA has emerged as a promising counter‐agent. A landmark 2025 double‐blind, randomized, placebo‐controlled trial provided compelling clinical evidence. Amadi et al. [22] treated patients with type 2 diabetes (T2D) with a GA‐rich phenolic fraction from *Anisopus mannii* (PhAM, 500 mg/day for 12 weeks). The treatment resulted in a significant decrease in HbA1c and fasting blood glucose levels compared to placebo. Mechanistically, the study proposed that GA directly modulates phosphofructokinase‐1 (PFK‐1), a critical rate‐limiting enzyme in glycolysis, thereby improving the cell's ability to metabolize glucose [22]. However, it is crucial to note that this study utilized a multi‐component phenolic fraction rather than purified gallic acid. While the results are promising, attributing the clinical benefits solely to GA requires further validation using pure compound formulations.

Complementing enzymatic modulation, GA targets nonenzymatic pathways essential for preventing diabetic complications. It significantly inhibits the formation of advanced glycation end‐products (AGEs), which are primary drivers of macrovascular damage. Furthermore, GA acts on incretin systems similar to modern diabetic pharmacotherapy (e.g., GLP‐1 receptor agonists). Research suggests GA can inhibit dipeptidyl peptidase‐IV (DPP‐IV), thereby prolonging the half‐life of endogenous GLP‐1 [23]. This dual action—enhancing insulin secretion via the GLP‐1 axis while preventing AGE‐mediated vascular damage—positions GA as a multi‐target candidate for metabolic syndrome.

In preclinical models, GA improves insulin sensitivity by activating the Akt and AMP‐activated protein kinase (AMPK) pathways. Activation of these pathways (e.g., in 3T3‐L1 adipocytes or C2C12 myotubes) promotes the translocation of glucose transporter type 4 (GLUT4) to the cell membrane, enhancing glucose uptake from the bloodstream [10]. Perhaps one of the most sophisticated mechanisms has been elucidated in diabetic nephropathy (DN). A 2024 study by Lee et al. [19] used a db/db mouse model of glucolipotoxicity‐induced DN. They found that GA treatment (50 mg/kg) provided potent renal protection. Microarray analysis revealed that diabetic kidneys upregulate miR‐709, a microRNA that directly suppresses Nrf2 (the master antioxidant transcription factor). GA treatment was found to downregulate this pathological expression of miR‐709. By inhibiting this inhibitor, GA “unleashed” Nrf2, allowing it to activate the antioxidant response element (ARE) and increase the production of protective enzymes like SOD and CAT, thereby shielding the kidney from oxidative stress and fibrosis [19].

### 3.2 Ameliorating Dyslipidemia and Atherosclerosis

GA also directly improves the liver's ability to clear low‐density lipoprotein (LDL) cholesterol. The LDL Receptor (LDLR) on hepatocytes is crucial for this process. A 2024 study by Zhang et al. [24] using HepG2 liver cells demonstrated that GA (at 50–200 µM) promotes LDL uptake by increasing LDLR accumulation through a dual mechanism. First, GA upregulates LDLR expression by stimulating the epidermal growth factor receptor (EGFR)–extracellular signal‐regulated kinase (ERK1/2) signaling pathway, which promotes LDLR gene transcription. Second, GA inhibits LDLR degradation. It suppresses the expression of proprotein convertase subtilisin/kexin type 9 (PCSK9) (the enzyme that targets LDLR for destruction) by modulating two transcription factors: promoting forkhead box O3 (FOXO3) while suppressing hepatocyte nuclear factor‐1α (HNF1α). This multi‐pronged approach—boosting LDLR synthesis while blocking its destroyer, PCSK9—results in a significant net increase in functional receptors, enhancing LDL clearance and providing a strong anti‐atherosclerotic effect [24].

## 4 The Dichotomous Role of Gallic Acid in Oncology: From Antioxidant to Pro‐Oxidant

The role of GA in cancer is a compelling example of context‐dependent pharmacology. While a protective antioxidant in healthy tissues, it can transform into a potent pro‐oxidant within the tumor microenvironment, selectively targeting cancer cells [6].

### 4.1 Pleiotropic Mechanisms of Antineoplastic Action

GA and its derivatives exhibit antineoplastic effects against diverse cancers [4, 25]. This activity is often selective; for example, Park [26] demonstrated that GA exhibits selective cytotoxicity, inhibiting the growth of HeLa cervical cancer cells significantly more effectively than human umbilical vein endothelial cells (HUVECs).

This selectivity stems from GA's ability to exploit the high intrinsic oxidative stress of cancer cells. Recent syntheses highlight that this context‐dependent cytotoxicity is a hallmark of GA's redox behavior, where it functions as a pro‐oxidant to deplete intracellular glutathione (GSH) and generate ROS specifically in the tumor microenvironment [12]. In pancreatic cancer, Kim et al. [27] demonstrated that GA (50–100 µM) induces a surge of intracellular ROS in PANC‐1 and MIA PaCa‐2 cells, triggering two cell death pathways: Endoplasmic Reticulum (ER) Stress (via the PERK/CHOP axis) and activation of the p38 MAPK pathway. Crucially, however, ROS generation is not the sole driver. Specifically, You and Park [28] demonstrated in Calu‐6 and A549 lung cancer cells that GA‐induced apoptosis is inextricably linked to both ROS elevation and GSH depletion. Notably, the antioxidant N‐acetyl‐cysteine (NAC) intensified GA‐induced cytotoxicity, a phenomenon tightly correlated with exacerbated GSH depletion, suggesting that disrupting cellular redox homeostasis—rather than ROS generation alone—is the critical mechanism. Extending this finding, You et al. [29] and Park [26, 30] definitively showed that in HeLa cervical cancer cells and pulmonary endothelial cells, this GSH depletion is the primary driver of cell death, occurring independently of ROS level fluctuations.

GA's action is pleiotropic, interfering with multiple stages of cancer. It inhibits metastasis by downregulating Matrix metalloproteinases (MMPs); Chen et al. [31] showed that non‐toxic doses of GA (20–40 µM) suppressed TPA‐induced MMP‐9 expression in MCF‐7 breast cancer cells. It also suppresses the Wnt/β‐catenin pathway in gastric lesions [32] and inhibits angiogenesis in glioma cell models [33].

### 4.2 Synergistic Potential With Conventional Therapies

GA shows remarkable synergy with targeted therapies. In Chronic Myeloid Leukemia (CML), driven by the BCR::ABL1 oncoprotein, Xiang et al. [34] found GA effectively inhibits proliferation in CML cell lines (K562, LAMA84) and, importantly, in primary CD34^+^ cells from CML patients (at 40–160 µM). A key mechanism identified was the inhibition of mitochondrial respiration. Most significantly, when combined with tyrosine kinase inhibitors (TKIs) like imatinib, GA showed powerful synergy (combination index < 1), suggesting it could be a powerful adjunctive therapy to enhance TKI efficacy [34]. These diverse actions are summarized in the expanded Table 1.

**TABLE 1:** Summary of key antineoplastic mechanisms of gallic acid.

Mechanism	Description	Model system(s)	GA Dose/treatment	Key molecular events/pathways	Key outcome(s)	Reference(s)
GSH‐dependent apoptosis	Triggers cell death driven by severe GSH depletion rather than ROS flux alone.	Pancreatic (PANC‐1); cervical (HeLa); lung (Calu‐6); endothelial (HUVEC and CPAEC)	30–100 µM (in vitro)	↑ROS, ↓GSH, ER stress (PERK/CHOP), p38 MAPK activation, caspase activation	Selective cytotoxicity in cancer cells; apoptosis induction	[26, 27, 28, 29, 30]
Cell cycle arrest	Halts uncontrolled cancer cell proliferation by stopping the cell division cycle.	Lung cancer cells (A549)	0–100 µM (in vitro)	Modulation of CDKIs (p21, p27); Inhibition of PI3K/Akt pathway	G2/M phase arrest; inhibition of proliferation	[35]
Inhibition of metastasis	Prevents cancer cells from migrating and invading surrounding tissues.	Breast cells (MCF‐7); gastric precancerous lesions (rat model)	20–40 µM (in vitro); 60 mg/kg (in vivo)	Downregulation of MMP‐9 (via EGFR/Src/Akt/Erk); inhibition of Wnt/β‐catenin	Reduced cell migration and invasion; alleviation of gastric lesions	[31, 32]
Anti‐angiogenesis	Inhibits the formation of new blood vessels that supply nutrients to tumors.	Glioma cells (U87, U251); HUVECs	40–80 µg/mL (in vitro)	Suppression of VEGF expression and secretion	Inhibition of tube formation; reduced tumor microvessel density	[33]
Synergy with targeted therapy	Inhibits mitochondrial energy production, enhancing the efficacy of standard‐of‐care TKIs.	CML cells (K562, LAMA84); Primary CML CD34^+^ cells	40‐160 µM (GA); combination with Imatinib	Inhibition of mitochondrial respiration; modulation of BCR::ABL1 signaling	Synergistic inhibition of CML cell growth (combination index < 1)	[34]

Abbreviations: ROS, reactive oxygen species; GSH, glutathione; ER, endoplasmic reticulum; MAPK, mitogen‐activated protein kinase; CDKIs, cyclins and cyclin‐dependent kinase inhibitors; Akt, protein kinase B; MMPs, matrix metalloproteinases; TKI, tyrosine kinase inhibitor; CML, chronic myeloid leukemia; HUVEC, human umbilical vein endothelial cell; VEGF, vascular endothelial growth factor; CPAEC, calf pulmonary arterial endothelial cell.

### 4.3 Safety Considerations and Pharmacodynamic Interactions

While the pro‐oxidant strategy is effective against tumors, safety concerns regarding high‐dose GA exposure remain. Although generally considered safe, sustained high concentrations of GA could potentially induce oxidative stress in normal tissues with low antioxidant capacity. Furthermore, the synergy with TKIs described by Xiang et al. [34] warrants caution regarding pharmacodynamic interactions. Since GA modulates transporters and enzymes that may overlap with TKI metabolism, rigorous pharmacokinetic monitoring is essential in combinatorial regimens to prevent unexpected toxicity or altered therapeutic windows.

## 5 Advanced Neuropharmacology: From Neuroprotection to Ion Channel Modulation

The application of GA in neurological disorders represents one of the most exciting frontiers of its research. The latest findings reveal a sophisticated mode of action, repositioning GA as a specific, pharmacologically active small molecule.

### 5.1 Countering Neurodegeneration

In Alzheimer's Disease (AD), GA is emerging as a multi‐target agent. A critical link exists between metabolic dysfunction and neurodegeneration, often termed “Type 3 Diabetes”, where insulin resistance in the brain drives cognitive decline. GA's ability to improve glucose metabolism (as discussed in Section 3) and possibly enhance GLP‐1 signaling thus offers a direct neuroprotective benefit.

Building on this, a groundbreaking 2024 study by Padhi et al. [36] detailed the synthesis of GCTR, a specific hybrid molecule chemically derived from the GA scaffold, designed to combat two distinct pathological processes:

1. Ferroptosis: An iron‐dependent cell death contributing to neuronal loss in AD. The GCTR molecule was shown to be the first synthetic small molecule to effectively combat ferroptosis by both restoring the enzymatic activity and increasing cellular levels of its master regulator, Glutathione Peroxidase 4 (GPX4), in neuronal cell models.
2. Amyloid toxicity: While natural GA has limitations in penetrating the blood‐brain barrier, the synthetic GCTR derivative was found to disrupt the iron (Fe^3^^+^)‐induced liquid‐liquid phase separation (LLPS) of the tau protein. LLPS is a critical early step in the formation of pathological tau aggregates (NFTs). By preventing this initial phase separation, this GA‐based pharmacophore strikes at the very beginning of tangle formation [36].

In Parkinson's disease (PD) models, GA also shows significant neuroprotection. Rangsinth et al. [37] used the neurotoxin 6‐hydroxydopamine (6‐OHDA) to induce toxicity in dopaminergic SH‐SY5Y cells. Pre‐treatment with GA (25–50 µM) protected the cells by scavenging ROS and, more specifically, by upregulating critical pro‐survival signaling pathways, including the Akt/mTOR and MEK/ERK pathways.

### 5.2 A Novel Frontier in Channelopathies

Perhaps the most paradigm‐shifting discovery is GA's role in channelopathies like Episodic Ataxia 1 (EA1), an inherited disorder caused by loss‐of‐function mutations in the voltage‐gated K^+^ channel subtype 1.1 (Kv1.1). Guided by traditional First Nations medicine, Manville et al. [38] found that GA is a potent modulator of Kv1.1. In *Xenopus* oocytes and mammalian cells expressing EA1‐linked mutant channels (e.g., I262T, V408A), GA (at 10–100 µM) directly rescued channel function by enhancing the potassium current. Molecular dynamics simulations revealed a precise mechanism: GA binds directly to the extracellular S1‐S2 linker of the channel's voltage‐sensing domain (VSD), inducing a conformational change that allosterically facilitates channel opening [38]. This discovery is profoundly important, as it demonstrates that GA can act as a classical positive allosteric modulator, elevating it to a lead compound for rational drug design targeting specific ion channels.

## 6 Overcoming Pharmacokinetic Hurdles and Future Applications

### 6.1 Overcoming Pharmacokinetic Hurdles With Advanced Formulations

Despite compelling preclinical bioactivity, the clinical translation of GA is significantly hindered by its pharmacokinetics. As a BCS Class III compound, GA's high aqueous solubility and low membrane permeability lead to poor oral bioavailability [39]. Its high aqueous solubility and low membrane permeability classify it as a biopharmaceutics classification system (BCS) Class III compound [12]. Although this sub‐optimal pharmacokinetic profile impedes clinical translation, strategic interventions such as molecular derivatization and nanotechnology‐based delivery architectures are being evaluated to circumvent these physiological barriers [12, 39].

As summarized in a 2021 review by Bai et al., pharmacokinetic studies in rodents and humans show that orally administered GA is rapidly absorbed (peak plasma concentration < 1 h) but also rapidly metabolized (via methylation and glucuronidation) and eliminated, with a very short half‐life, severely limiting its systemic exposure and efficacy.

To surmount this, researchers are pursuing two main strategies: medicinal chemistry and novel drug delivery systems (NDDS). Medicinal chemistry aims to create derivatives (e.g., alkyl esters, hybrids) with improved lipophilicity to enhance membrane crossing [4, 40], as seen with the GCTR hybrid in AD models [36]. Concurrently, NDDS research focuses on encapsulating GA within nanocarriers (liposomes, polymeric micelles, nanoparticles) to protect it from degradation, improve its solubility profile, and enhance transport across membranes [4, 39].

### 6.2 Emerging Applications: Gallic Acid as a Bioactive Material

The most advanced research is pushing the boundaries of how GA is utilized, moving from “delivering GA” to “building with GA”. Recent 2025 findings demonstrate GA's versatility as a structural and functional component in advanced biomaterials. In tissue engineering and wound care, GA's intrinsic properties are being leveraged to create multifunctional scaffolds. Guo et al. [41] constructed an antibacterial and antioxidant adherent sponge by utilizing GA‐mediated assembly of fibrous clay in collagen, demonstrating rapid hemostasis for arterial bleeding control. Similarly, Zhao et al. [42] developed an enzyme‐induced, thermally shape‐adaptive gelatin‐GA hydrogel that effectively manages infected skin wounds through photothermal therapy and ROS scavenging.

This “active material” concept extends to internal medicine. A prime example is an injectable hydrogel for osteoarthritis (OA), where Li et al. [43] chemically grafted GA onto a gelatin backbone. In this system, GA functions as an “active excipient”, providing intrinsic ROS‐scavenging and anti‐inflammatory activity (by regulating the PI3K/AKT pathway). Building on this foundation, He et al. [44] engineered injectable microspheres containing a gallic acid‐magnesium metal‐organic framework (GA‐Mg MOF). This sophisticated system not only delivered sustained anti‐inflammatory effects but also alleviated cartilage degeneration in osteoarthritis models. Furthermore, Zhang et al. [45] designed ultrasmall, self‐assembled GA‐based natural defense networks specifically to inhibit calcium oxalate crystal aggregation, offering a novel therapeutic strategy for nephropathies (kidney stones).

An even more fundamental use is in GA‐based metal‐phenolic networks (MPNs). These smart materials are formed by the pH‐dependent self‐assembly of GA and metal ions (e.g., Fe^3^^+^) [18]. These networks can form functional coatings for environmental remediation, food packaging, and biomedical sensors. This evolution—from GA as an active pharmaceutical ingredient (API) to a bioactive material component—opens a vast design space for next‐generation therapies.

## 7 Conclusion and Future Outlook

### 7.1 Synthesis of Findings

Gallic acid has evolved from a simple antioxidant to a highly specific pharmacological agent and a versatile biomaterial. This review charts this evolution, highlighting its role as an upstream regulator of oxidative stress (via Nrf2) and inflammation (via NF‐κB); its function as a metabolic reprogrammer (modulating PFK‐1, miR‐709, and PCSK9); its context‐dependent duality in oncology (acting as a pro‐oxidant and TKI‐synergist); and its advanced role as a specific molecular modulator in neurology (inhibiting ferroptosis/LLPS and directly gating ion channels like Kv1.1). Crucially, recent advancements demonstrate its emerging capability as a functional building block for advanced therapeutic materials, such as MOFs and hemostatic sponges, specifically targeting wound healing and tissue regeneration.

**FIGURE 3:**
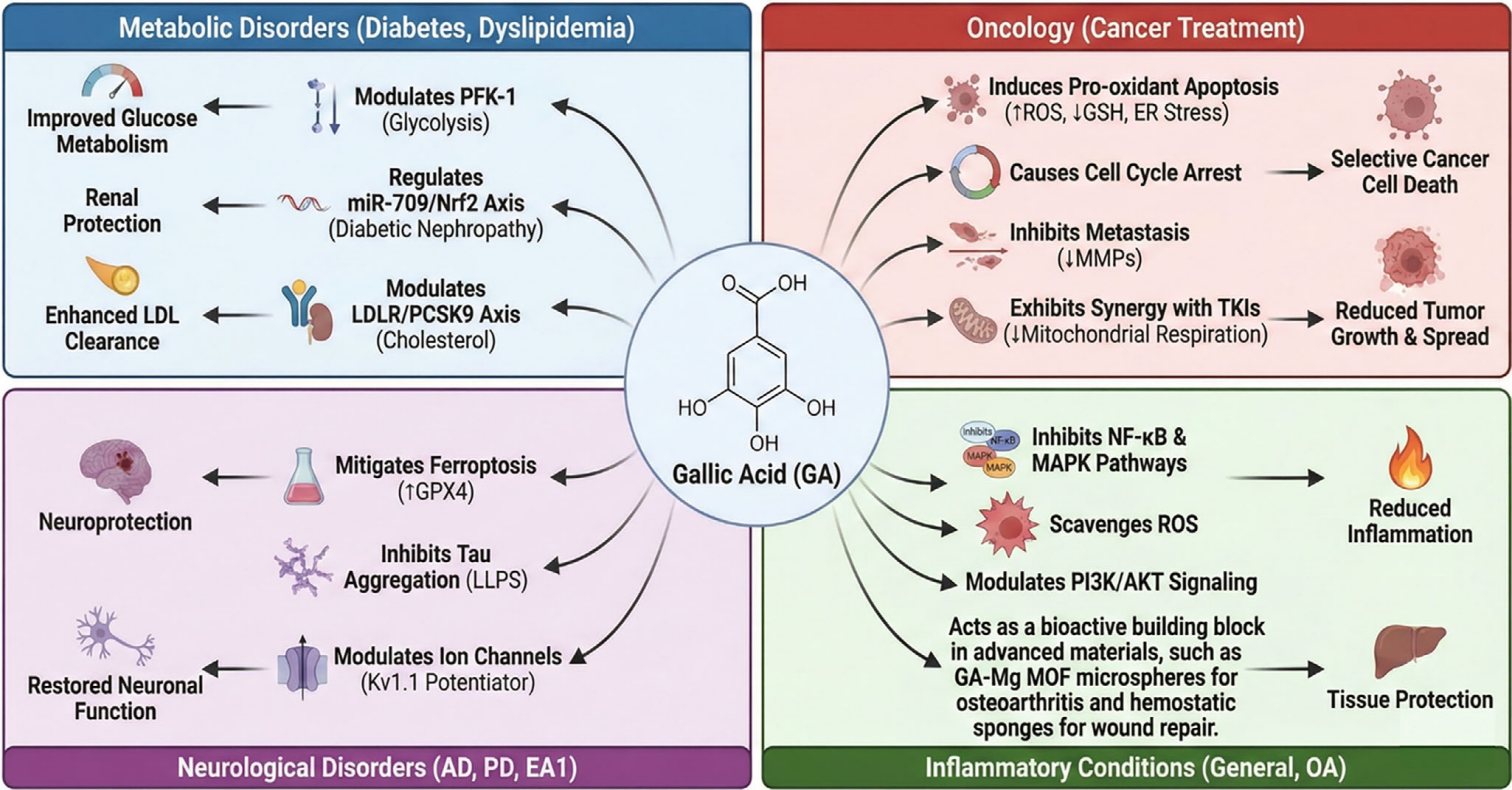
Comprehensive schematic of gallic acid's multifaceted mechanisms in disease pathogenesis (preclinical and clinical findings). This diagram synthesizes the diverse therapeutic actions of gallic acid (GA) across four major pathological categories, based on recent preclinical and clinical evidence. Metabolic Disorders (top‐left): GA acts as a metabolic modulator, regulating PFK‐1 in diabetes, the miR‐709/Nrf2 axis in diabetic nephropathy, and the LDLR/PCSK9 pathway to enhance LDL cholesterol clearance. Oncology (Top‐Right): In cancer treatment, GA functions as a selective pro‐oxidant to induce apoptosis, arrest the cell cycle, inhibit metastasis (via MMPs), and exhibit synergy with tyrosine kinase inhibitors (TKIs) by inhibiting mitochondrial respiration. Neurological disorders (bottom‐left): GA demonstrates advanced neuroprotective effects by mitigating ferroptosis (via rescuing GPX4 activity), inhibiting tau aggregation (specifically liquid‐liquid phase separation, LLPS), and acting as a direct positive allosteric modulator of Kv1.1 ion channels. Inflammatory conditions (bottom‐right): GA exerts broad anti‐inflammatory activity by suppressing the NF‐κB and MAPK signaling pathways and directly scavenging reactive oxygen species (ROS), and modulating PI3K/AKT signaling for tissue protection, and acts as a bioactive building block in advanced materials, such as GA‐Mg MOF microspheres for osteoarthritis and hemostatic sponges for wound repair.

The diverse, specific mechanisms discussed are brought together in Figure 3 and Table 2, which provide a comprehensive schematic and a detailed summary, respectively, of GA's multifaceted therapeutic actions.

**TABLE 2:** Summary of gallic acid's mechanistic actions in major disease categories.

Disease category	Pathological process targeted	Model system(s)	GA dose/treatment	Primary mechanism of GA action	Key reference(s)
Diabetes mellitus (T2D)	Hyperglycemia, glycolytic dysregulation	Human clinical trial (T2D patients)	500 mg/day (PhAM fraction)	Modulates phosphofructokinase‐1 (PFK‐1) to improve glucose metabolism.	[22]
Diabetic nephropathy	Glucolipotoxicity, oxidative stress	db/db Mice (in vivo)	50 mg/kg/day	Downregulates miR‐709 to unleash Nrf2, boosting endogenous antioxidant enzymes (SOD, CAT).	[19]
Atherosclerosis	LDL cholesterol accumulation	HepG2 cells (in vitro)	50–200 µM	Increases LDLR expression (via EGFR‐ERK) and inhibits LDLR degradation (by suppressing PCSK9).	[24]
Oncology	Proliferation, survival, metastasis	Various cancer cell lines (PANC‐1, HeLa, A549, K562) (in vitro)	40–160 µM	Induces selective pro‐oxidant apoptosis (↑ROS, ↓GSH); Arrests cell cycle; Acts synergistically with TKIs.	[26, 27, 29, 34, 35]
Alzheimer's disease	Ferroptosis and tau aggregation	Neuronal cell lines (in vitro)	10–20 µM (GCTR hybrid)	Rescues GPX4 activity to inhibit iron‐dependent cell death; Disrupts iron‐induced tau liquid‐liquid phase separation (LLPS).	[36]
Episodic ataxia 1	Ion channel dysfunction (Kv1.1)	Xenopus oocytes and Mammalian cells (in vitro)	10–100 µM	Binds directly to the Kv1.1 VSD (S1‐S2 linker), acting as a positive allosteric modulator to rescue channel function.	[38]
Nephropathies (kidney stones)	Calcium oxalate crystal formation	In vitro/in vivo models	Self‐assembled GA networks	Forms ultrasmall natural defense networks to inhibit crystal aggregation and cell injury.	[45]
Osteoarthritis	Joint inflammation and cartilage degradation	Rat model (in vivo)	Injectable hydrogel/microspheres	Acts as a bioactive material. GA‐Mg MOF microspheres alleviate cartilage degeneration.	[43, 44]
Wound healing and hemostasis	Bleeding, bacterial infection	In vivo (arterial bleeding, infected skin)	GA‐based sponge/hydrogel	Facilitates hemostasis via fibrous clay assembly; provides photothermal/antibacterial effects.	[41, 42]

Abbreviations: PFK‐1, phosphofructokinase‐1; Nrf2, nuclear factor erythroid 2‐related factor 2; LDLR, low‐density lipoprotein receptor; EGFR, epidermal growth factor receptor; ERK1/2, extracellular signal‐regulated kinase; PCSK9, proprotein convertase subtilisin/kexin type 9; ROS, reactive oxygen species; GSH, glutathione; TKI, tyrosine kinase inhibitor; GPX4, glutathione peroxidase 4; Kv1.1, voltage‐gated K+ channel subtype 1.1; VSD, voltage‐sensing domain; PhAM, phenolic acid‐rich fraction from *Anisopus mannii*; GA‐Mg, gallic acid‐magnesium; MOF, metal‐organic framework.

### 7.2 Current Limitations and Future Directions

Despite compelling preclinical data, the widespread clinical application of GA remains an unrealized goal. The primary hurdles are twofold:

1. Pharmacokinetics and Bioavailability: As a BCS Class III compound, GA's low permeability and rapid metabolism remain the single greatest barrier [12, 16, 39]. Advanced NDDS and derivative development are promising but require optimization for safety, scalability, and cost.
2. Clinical Validation: There is a critical lack of robust human clinical trials. The 2025 trial on the PhAM fraction is a significant step forward [22], but more large‐scale trials are needed to confirm efficacy, establish dosing, and understand human metabolism for specific disease indications.

Finally, the translation of GA‐based biomaterials from bench to bedside faces distinct challenges, particularly regarding scalability and standardization. The synthesis of uniform GA‐metal networks and the maintenance of batch‐to‐batch consistency in natural‐derived hydrogels present significant regulatory hurdles. Therefore, establishing Good manufacturing practice (GMP)‐compliant protocols is a prerequisite to securing regulatory approval for these advanced bioactive materials.

To overcome these barriers and expand the therapeutic horizon, future research must proceed along parallel tracks: medicinal chemistry to optimize ADME properties, advanced biomaterial engineering to exploit GA's structural chemistry for local delivery systems (e.g., injectable microspheres or shape‐adaptive hydrogels), and rigorous clinical research. A high priority should be placed on validating GA's emerging molecular targets—specifically, Kv1.1 and GPX4—in human trials. The evidence synthesized here strongly suggests that GA is not merely an antioxidant, but a versatile molecular tool with a profound therapeutic potential that is only just beginning to be fully harnessed.

## Funding

This work was supported by the “Research Base Construction Fund Support Program” funded by Jeonbuk National University in 2024.

## Conflicts of Interest

The author declares no conflicts of interest.

## Use of Generative AI and AI‐Assisted Technologies in the Writing Process

An Artificial Intelligence (AI) language model was utilized solely as a tool for language refinement, including grammar and spelling checks, and for summarizing provided reference materials. The author was solely responsible for the conception of the study, literature sourcing and analysis, data interpretation, and the drafting of the entire manuscript. The author assumes full responsibility for the intellectual content, accuracy, and originality of this work.

## Data Availability

The author has nothing to report.
